# Overcoming the Hurdle With Delicate Balance: TAVR in Prior Mechanical Mitral Prosthesis

**DOI:** 10.1016/j.jaccas.2025.105848

**Published:** 2025-10-22

**Authors:** Prashant Dwivedi, Kush Mehrotra

**Affiliations:** Department of Interventional Cardiology, Eternal Hospital, Jaipur, India

**Keywords:** aortic valve, mitral valve, valve replacement

**Figure:**
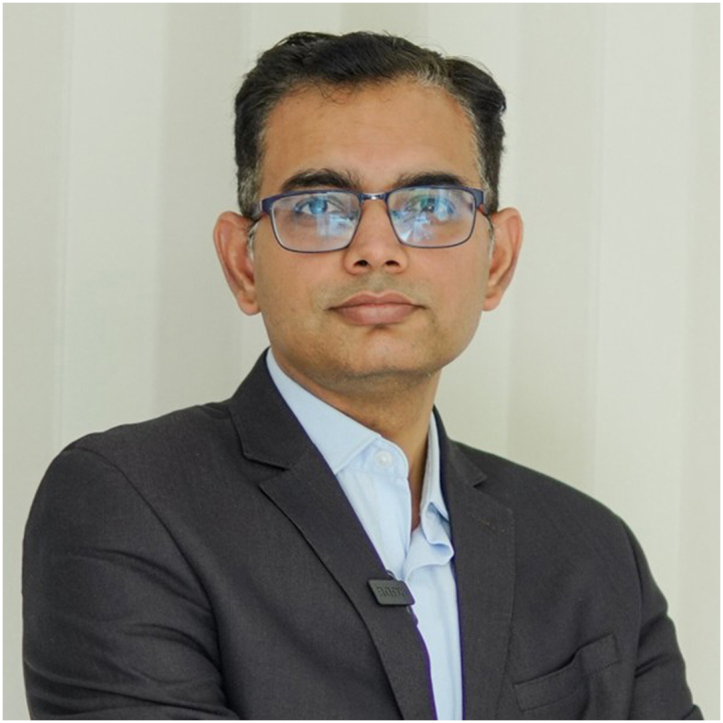

Since the inception of transcatheter aortic valve replacement (TAVR) in 2002, initially performed in a patient with severe aortic stenosis with prohibitive surgical risk, the indications for this procedure have expanded gradually to include patients with intermediate or low surgical risk. Patients with previous surgical mitral valve repair or replacement were excluded from earlier TAVR clinical trials.

In a real world scenario, a subset of patients will undergo mitral valve replacement (MVR) with a mechanical or bioprosthetic valve for various reasons. Approximately three-fourths of patients who undergo MVR develop aortic valve disease during follow-up, with 5% of them requiring aortic intervention in the future.^1^ For potential surgical aortic valve replacement candidates, previous cardiac surgery elevates the operative risk substantially, thus making TAVR a reasonable alternative.

In this issue of *JACC: Case Reports*, Garg et al^2^ present their case series of TAVR performed in 4 patients with rheumatic heart disease and previous mechanical mitral prostheses (MMPs), with an average aortomitral distance of 3.25 mm. Garg et al^2^ achieved 100% procedural and clinical success in this complex subset of patients.

TAVR in a patient with a previous MMP presents several technical challenges, such as the following: 1) a risk of interference between the new aortic transcatheter heart valve (THV) and the previous MMP leaflet that leads to a stuck mechanical valve; 2) a risk of mechanical valve dehiscence with a balloon-expandable valve (BEV) or a predilation and postdilation balloon; and 3) a risk of new aortic THV migration or embolization (Figure 1).

**Figure 1:**
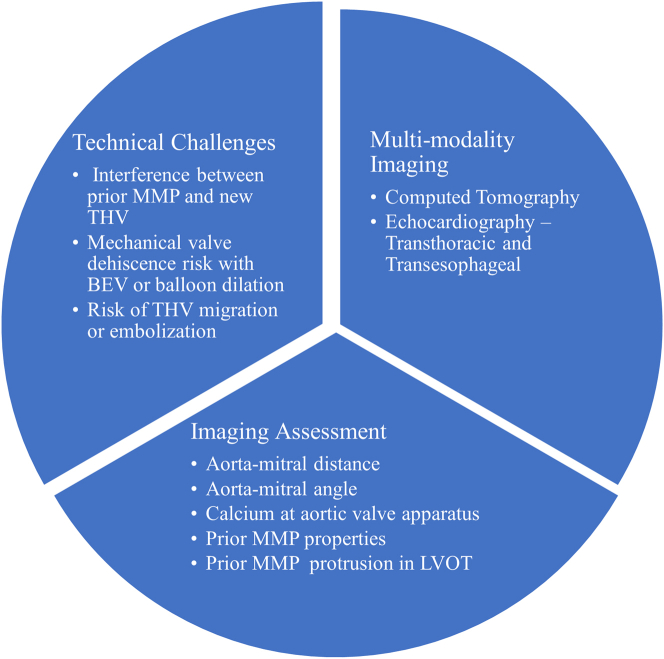
Challenges and Imaging in Transcatheter Aortic Valve Replacement in Patients With a Previous MMP BEV = balloon-expandable valve; LVOT = left ventricular outflow tract; MMP = mechanical mitral prosthesis; THV = transcatheter heart valve.

To overcome these challenges, detailed evaluation and meticulous planning combined with expert execution are of paramount importance. Multimodality imaging with contrast-enhanced computed tomography and echocardiography (transthoracic or transesophageal) forms the foundation of technical success.

In this subset of patients, apart from routine computed tomographic TAVR assessment, we need to look for the following: 1) aortomitral distance; 2) aortomitral angle; 3) calcium at the aortic valve apparatus; 4) properties of the previous MMP; and 5) protrusion of the previous MMP into the left ventricular outflow tract (LVOT) (Figure 2).

**Figure 2:**
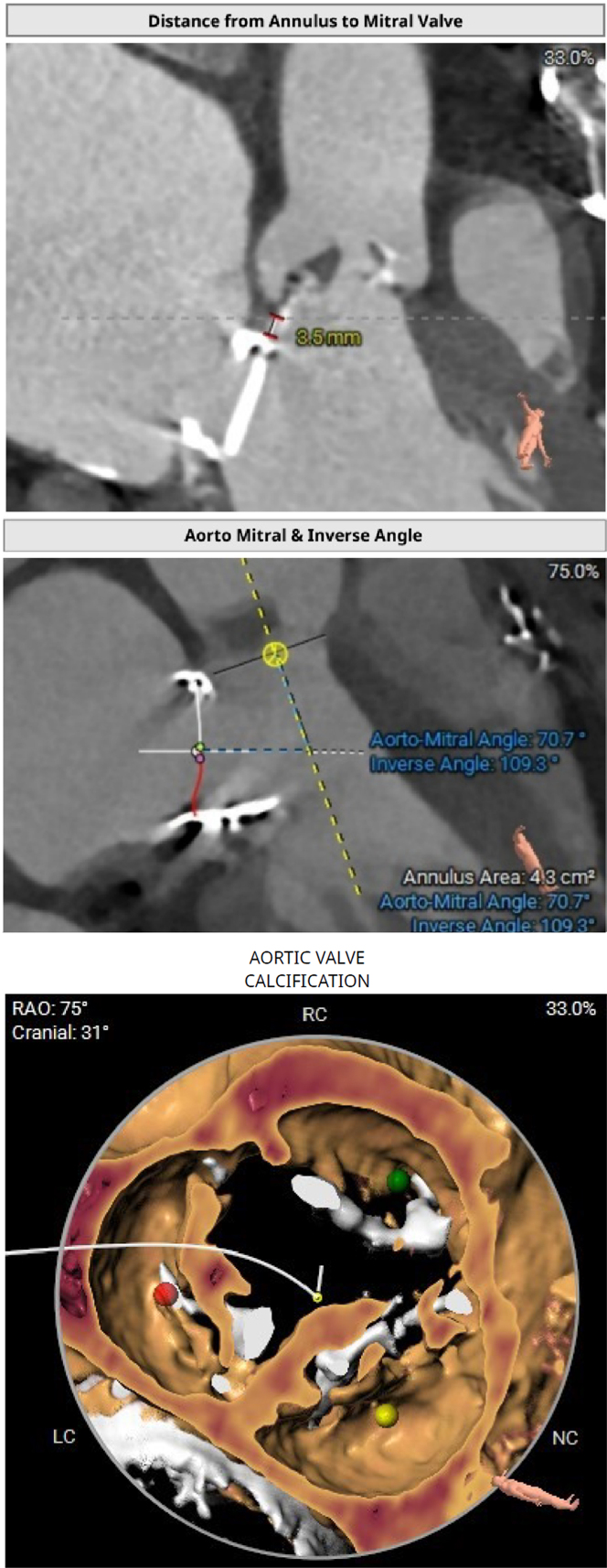
Imaging in Transcatheter Aortic Valve Replacement in Patients With a Previous Mechanical Mitral Prosthesis LC = left coronary cusp; NC = noncoronary cusp; RAO = right anterior oblique; RC = right coronary cusp.

MMP may cause blooming artifacts in a few of these cases, thus affecting accurate aortomitral distance measurement and aortic annular sizing. In such cases, 3-dimemsional transesophageal echocardiography is used as a complementary imaging strategy.

To consider an aortomitral distance of more than 7 mm as safe point for TAVR in patients with a previous MMP is arbitrary and is based on the Spanish registry^3^; however, there is no definite cutoff. If the aortomitral distance is <3 mm, then a BEV, which can be implanted at a higher position (90:10 vs 80:20), considering enough calcification at the aortic valve, is usually preferred. If the aortomitral distance is >3 mm, then either a BEV or a self-expandable valve (SEV) can be implanted. However, this distinction is subjective because many case reports have demonstrated safely implanted SEVs, even in patients with a shorter aortomitral distance. If the aortomitral distance is short, then the aortomitral angle assumes significance. The probability of interference with an MMP is high when the aortomitral angle is <90°; if the angle is >120°, then the probability of impingement is relatively low.^4^

In some patients with a previous MMP, the underlying cause is rheumatic or mixed (rheumatic and degenerative). These patients usually have thickened fibrotic leaflets and minimal calcification at the aortic valve complex. High implantation of a THV, often required in patients with mild annular calcium or a short aortomitral distance with a previous MMP, increases the risk of embolization because of inadequate annular anchoring and reduced aortic root support. Aortic THV interaction with a previous MMP protruding into the LVOT can lead to a “watermelon seeding” phenomenon and a potentially increased risk of embolization or migration.

The properties of previous MMPs, such as the type of valve (caged ball, single leaflet, or bileaflet), their profile height during both open and closed positions, and their surgical implantation method (anatomical or anti-anatomical), are important to assess the leaflet protrusion into the LVOT and to avoid further mechanical interference with the aortic THV. An anatomically implanted bileaflet valve (oriented like an anterior and posterior mitral valve) or an antianatomically implanted single tilting disc valve (major orifice opening to the LVOT) carries a risk of impingement and a stuck THV.^4^

Following wire crossing of the aortic valve, assessment with extreme fluoroscopic angulations (eg, left anterior oblique cranial or right anterior oblique cranial) should be performed to exclude inadvertent passage through the mitral apparatus. Additionally, during pre- or postdilation, balloon inflations should be minimized and positioned eccentrically toward the aorta to reduce the risk of injury to a preexisting MMP. Optimal vascular access and closure assume heightened significance in this challenging clinical context because anticoagulant agents need to be initiated again as soon as possible after TAVR.

Most imperative is to appreciate that these TAVR procedures demand individualized planning and should be performed only by experienced operators. In patients with previous MMPs, a 3-armed prospective comparative study evaluating surgical aortic valve replacement vs TAVR with a BEV vs TAVR with an SEV would be welcome to formulate future treatment recommendations and guidelines.

## Funding Support and Author Disclosures

The authors have reported that they have no relationships relevant to the contents of this paper to disclose.
